# Systemic Inflammation and Risk of Thromboembolism, Mortality, and Treatment Response in Patients With Cancer Receiving Immune Checkpoint Inhibitors

**DOI:** 10.1111/eci.70243

**Published:** 2026-07-24

**Authors:** Leyla Ay, Ingrid Pabinger, Matthias Preusser, Anna Sophie Berghoff, Christoph Hoeller, Cihan Ay, Florian Moik

**Affiliations:** ^1^ Karl Landsteiner Institute of Lung Research and Pulmonary Oncology Vienna Austria; ^2^ Department of Respiratory and Critical Care Medicine Klinik Floridsdorf, Vienna Healthcare Group Vienna Austria; ^3^ Division of Hematology and Hemostaseology, Department of Medicine I Medical University of Vienna Vienna Austria; ^4^ Division of Oncology, Department of Medicine I Medical University of Vienna Vienna Austria; ^5^ Christian Doppler Laboratory for Personalized Immunotherapy, Department of Medicine I Medical University of Vienna Vienna Austria; ^6^ Department of Dermatology Medical University of Vienna Vienna Austria; ^7^ Division of Oncology, Department of Internal Medicine Medical University of Graz Graz Austria

## Abstract

**Background:**

Immune checkpoint inhibitors (ICI) are widely used in cancer therapy, but biomarkers for thromboembolic risk, treatment response, and survival remain limited. We evaluated the association of systemic inflammatory indices with these clinical outcomes.

**Methods:**

In this retrospective cohort study, 580 ICI‐treated patients at the Medical University of Vienna, Austria, were included. Inflammatory indices including neutrophil‐lymphocyte‐ratio (NLR), platelet‐lymphocyte‐ratio (PLR), lymphocyte‐monocyte‐ratio (LMR), systemic immune‐inflammation‐index (SII) and C‐reactive‐protein‐albumin‐ratio (CAR) were calculated at ICI‐start and longitudinally within 3 months. Co‐primary outcomes were risk of venous thromboembolism (VTE), assessed using competing risk analysis, and overall‐ and progression‐free‐survival (OS/PFS), evaluated with Cox regression, whereas longitudinal biomarker dynamics were analysed using time‐dependent analyses.

**Results:**

Higher baseline NLR (HR 1.65, 95% CI: 1.28–2.12), CAR (HR 1.99, 95% CI: 1.67–2.38), and SII (HR 1.18, 95% CI: 1.00–1.39) were independently associated with shorter OS, while higher LMR (HR 0.44, 95% CI: 0.30–0.67) was associated with longer survival. For PFS, elevated NLR (HR 1.29, 95% CI: 1.05–1.57), higher PLR (HR 1.18, 95% CI: 1.00–1.39), lower LMR (HR 0.57, 95% CI: 0.43–0.70), and higher CAR (HR 1.42, 95% CI: 1.28–1.62) predicted poorer outcomes. None of the indices at treatment start were associated with VTE, yet doubling of levels within 3 months indicated higher VTE risk for PLR (sub‐distribution hazard ratio [SHR]: 3.03 95% confidence interval [CI]: 1.05–8.08) and a borderline‐significant association for CAR (SHR: 2.02, 95% CI: 0.89–4.60).

**Conclusion:**

We found that selected inflammatory indices were associated with poor OS and PFS in ICI‐treated patients, and longitudinal dynamics might identify patients at higher risk for VTE.

## Graphical abstract

In patients with cancer receiving immune checkpoint inhibitors, systemic inflammatory indices measured at treatment initiation and during the first 3 months were associated with treatment response, survival, and thromboembolism, supporting their potential utility for risk stratification.

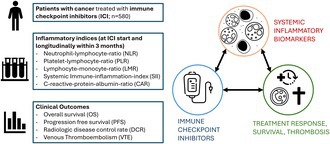

## 1 Introduction

Immune checkpoint inhibitors (ICI) are increasingly used in patients with cancer and have transformed the clinical course of disease for several tumour types [1]. ICI are characterized by sustained treatment responses and survival in a subset of patients; however, many patients do not derive benefit [1]. Although various prognostic and predictive biomarkers for ICI‐benefit have been evaluated in patients with cancer, there is still an unmet medical need for refined prediction of treatment response patterns and survival [2, 3]. Further, venous thromboembolism (VTE) is a frequent complication in patients treated with ICI, with over 10% of patients developing VTE within 12 months after ICI initiation [4, 5]. In the general population of patients with cancer, several risk factors and biomarkers for VTE have been identified, leading to the development of various risk assessment models aimed at stratifying VTE risk and identifying patients who may benefit from primary thromboprophylaxis [6, 7]. Interestingly, established risk assessment models including the Khorana score reportedly underperform in the setting of ICI therapy, and data on risk factors and biomarkers for VTE risk specifically in patients treated with ICI remain scarce [8].

Different blood‐cell based inflammatory indices have been identified in the past as robust biomarkers of adverse outcomes in patients with cancers in different clinical scenarios [9, 10, 11]. They include cell‐based indices such as the neutrophil‐lymphocyte‐ratio (NLR), the lymphocyte‐monocyte‐ratio (LMR), the platelet‐lymphocyte‐ratio (PLR), and the Systemic Immune‐Inflammation Index (SII), and protein‐based indices such as the C‐reactive‐protein‐albumin‐ratio (CAR). Currently, comprehensive data on the association of cell‐based inflammatory indices with treatment response and survival in patients with cancer treated with ICI are scarce, and their association with the risk of VTE during ICI‐therapy has not been evaluated. The aim of this study was to explore the association between baseline and longitudinal levels of inflammatory indices with treatment response, survival, and risk of VTE in ICI‐treated patients.

## 2 Methods

### 2.1 Study Population and Study Procedures

We conducted a single‐center retrospective cohort study at the Vienna General Hospital/Medical University of Vienna, Vienna, Austria. The study was approved by the institutional ethics committee (number 2213/2019; ethik-kom@meduniwien.ac.at). Detailed descriptions of the study procedures have been published previously [4]. This study was conducted and reported in accordance with the Strengthening the Reporting of Observational Studies in Epidemiology (STROBE) guidelines, and a completed STROBE checklist is provided as a File S1.

The study cohort comprised consecutive adult patients (≥ 18 years of age) with histologically confirmed malignancies who received at least one dose of an approved ICI (nivolumab, pembrolizumab, ipilimumab, atezolizumab, or avelumab) between January 2015 and November 2018. Eligible patients were identified through the institutional pharmacy prescription database. Data collection was performed through a comprehensive review of electronic medical records. Data was captured longitudinally from the initiation of ICI therapy until death, loss to follow‐up, or the end of the observation period.

The primary endpoints were venous thromboembolism (VTE), overall survival (OS), progression‐free survival (PFS), and disease control rate (DCR). VTE was defined as objectively confirmed and independently adjudicated symptomatic/incidental deep vein thrombosis, pulmonary embolism, splanchnic vein thrombosis, and fatal pulmonary embolism [12]. OS was defined as the time from ICI start until all‐cause death. Radiologic treatment responses were determined based on radiological and clinical assessments according to institutional practice and Response Evaluation Criteria in Solid Tumours (RECIST) version 1.1 [13]. PFS was defined as the time from ICI‐initiation until disease progression or death. DCR was defined as the proportion of patients achieving complete response, partial response, or stable disease as best response according to RECIST [13]. The observation period began at initiation of ICI therapy and ended at the earliest of: (1) initiation of subsequent systemic antineoplastic therapy, (2) loss to follow‐up, or (3) 3 months after the last ICI administration, in accordance with established safety‐reporting intervals in ICI clinical trials [14].

### 2.2 Inflammatory Indices

Inflammatory indices were evaluated at baseline and longitudinally during the first 3 months of ICI treatment, including the NLR, PLR, LMR, SII, and CAR (Table S1). All biomarker data were derived from routine peripheral blood samples obtained as part of standard clinical care. Baseline biomarker values were defined as those obtained on the date of the first ICI administration or within the preceding 2 weeks, using the measurement closest to treatment initiation. Longitudinal measurements were collected from all available blood draws during the first 3 months of therapy. The inflammatory indices were calculated using standard formulas, with NLR defined as the ratio of absolute neutrophil to lymphocyte counts, PLR as the ratio of platelet to lymphocyte counts, LMR as the ratio of lymphocyte to monocyte counts, SII calculated as the product of platelet and neutrophil counts divided by the lymphocyte count, and CAR defined as the ratio of C‐reactive protein (CRP) to albumin levels. Doubling of biomarker levels during the first 3 months of therapy were calculated relative to intra‐individual baseline levels.

### 2.3 Statistical Analysis

The association between biomarkers and risk of VTE was evaluated using competing‐risk regression, treating death as a competing event. Sub‐distribution hazard ratios (SHRs) and 95% confidence intervals (CIs) were estimated using the Fine–Grey model. For OS/PFS, Cox proportional‐hazards regression was used to obtain hazard ratios (HRs) and 95% CIs. Competing risk cumulative incidence estimates for VTE and Kaplan–Meier curves for OS and PFS were generated, and differences between groups were assessed with the log‐rank test for OS/PFS and Grey's test for VTE. DCR was analysed using binary logistic regression to estimate odds ratios (ORs) and 95% CIs. For the analyses of biomarkers with risk of VTE, OS, PFS and DCR, we considered two exposure definitions separately: (1) baseline biomarker levels on a continuous scale, using log2‐transformation to achieve adequate biomarker distribution, with dichotomization of biomarkers at the respective median (high vs. low) for visualization and (2) an early biomarker change defined as an intra‐individual doubling of the baseline value within the first 3 months of treatment. Time‐dependent analyses were performed to assess longitudinal changes in biomarker levels, using a landmark approach comparing patients with an early doubling of levels within 3 months after ICI initiation to those without and the Mantel‐Byar‐test for between group comparisons.

Multivariable models were applied to adjust for potential confounders including age, sex, cancer type, and stage. In addition, VTE analyses were further adjusted for BMI, prior VTE, and baseline anticoagulation, while OS and PFS analyses additionally included line of systemic anticancer therapy. Missing data were handled using multiple imputation by chained equations (MICE). Twenty‐five imputed datasets were generated using an imputation model that included the outcomes, baseline co‐variates, and available biomarkers. Estimates from the imputed datasets were combined using Rubin's rules to produce pooled effect estimates and standard errors. All tests were two‐sided and a *p*‐value < 0.05 was considered statistically significant. Given the exploratory nature of the analyses, no formal adjustment for multiple testing was performed. All statistical analyses were performed with the commercially available package STATA 15.1 (Stata, Houston, TX).

## 3 Results

### 3.1 Study Population

Overall, 580 consecutive patients treated with ICI were included. The median age at treatment initiation was 64 years (interquartile range [IQR], 53–72), and 40.5% of patients were female. Most patients had an Eastern Cooperative Oncology Group (ECOG) performance status of 0–1 (91.4%), and the median Charlson comorbidity index was 8 (IQR, 7–9). The most frequent tumour types were malignant melanoma (*n* = 192, 33.1%), non–small cell lung cancer (*n* = 158, 27.2%), renal cell carcinoma (*n* = 57, 9.8%), head and neck squamous cell carcinoma (*n* = 43, 7.4%), and urothelial cancer (*n* = 25, 4.3%). Most patients had distant metastases at ICI start (stage IV, 89.1%).

The most frequently administered ICI agents were nivolumab (*n* = 254, 43.8%) and pembrolizumab (*n* = 235, 40.5%), followed by ipilimumab (*n* = 45, 7.8%), nivolumab + ipilimumab combination therapy (*n* = 27, 4.7%), atezolizumab (*n* = 15, 2.6%), and avelumab (*n* = 4, 0.7%). The median number of ICI cycles administered was 6 (IQR, 4–17; range, 1–76). Most patients had received prior systemic therapy, including chemotherapy in 331 (57.1%). Concomitant antineoplastic therapies during ICI treatment included targeted therapy (9.5%) and chemotherapy (3.5%). After ICI discontinuation, 170 patients (29.3%) received subsequent medical anticancer therapy. Detailed patient and treatment characteristics are summarized in Table 1. Levels of inflammatory indices at baseline and changes within 3 months are shown in the Table S1.

**TABLE 1:** Baseline characteristics of study cohort.

Variable	*n* (% missing)	Median [IQR] or count (%)
Demographics and clinical characteristics		
Age (years)	580 (0%)	64 [53–72]; range: 19–89
Female	580 (0%)	235 (40.5%)
BMI (kg/m^2^)	477 (17.8%)	24.5 [21.6–28.0]
ECOG	467 (19.5%)	0 [0–1]
ECOG 0–1	/	427 (91.4%)
ECOG ≥ 2	/	40 (8.6%)
Charlson comorbidity index	580 (0%)	8 [7–9]
Modified Charlson comorbidity index^a^	580 (0%)	2 [1–4]
History of VTE	580 (0%)	74 (12.8%)
History of VTE during current cancer disease	580 (0%)	54 (9.3%)
Continuous anticoagulation	580 (0%)	104 (17.9%)
Continuous platelet inhibitor therapy	580 (0%)	120 (20.7%)
Tumour specifics at inclusion		
Tumour type	580 (0%)	/
Melanoma		192 (33.1%)
Non‐small cell lung cancer		158 (27.2%)
Renal‐cell carcinoma		57 (9.8%)
Head and neck squamous cell carcinoma		43 (7.4%)
Urothelial		25 (4.3%)
Lymphoma/Myeloma		24 (4.1%)
Gynaecological		18 (3.1%)
Sarcoma		17 (2.9%)
Hepatocellular cancer		13 (2.2%)
Colorectal cancer		11 (1.9%)
Others		22 (3.8%)
Stage	534 (7.9%)	/
I–III	/	58 (10.9%)
IV	/	476 (89.1%)
Distant metastatic site	/	/
Lung	/	232 (40.0%)
Bone	/	132 (22.8%)
Liver	/	130 (22.4%)
Cerebral	/	72 (12.4%)
PD‐L1 (TPS)	172 (70.3%)	10 [0–60]
PD‐L1 negative	/	56 (32.6%)
MSI‐high		11 (1.9%)
Therapeutic management		
ICI agent	580 (0%)	/
Pembrolizumab	/	235 (40.5%)
Nivolumab	/	254 (43.8%)
Ipilimumab	/	45 (7.8%)
Atezolizumab	/	15 (2.6%)
Avelumab	/	4 (0.7%)
Ipilimumab + Nivolumab	/	27 (4.7%)
ICI treatment cycles	/	6 [4–17], range: 1–76
Treatment intent	/	/
(pseudo‐)neoadjuvant	/	1 (0.2%)
(pseudo‐)adjuvant	/	19 (3.3%)
Palliative	/	560 (96.6%)
Line of anticancer therapy	/	2 [1–3], range: 1–7
Prior systemic therapy	/	331 (57.1%)
Chemotherapy combination with ICI	/	20 (3.5%)
Targeted therapy combination with ICI	/	55 (9.5%)
Systemic therapy after ICI	/	170 (29.3%)

Abbreviations: BMI, body mass index; ECOG, Eastern Cooperative Oncology Group; ICI, immune checkpoint inhibitor; IQR, interquartile range; MSI, microsatellite instability; PD‐L1, programmed death‐ligand 1; TPS, tumour proportion score; VTE, venous thromboembolism.

^a^ Modified Charlson Comorbidity Index calculated without points for cancer variable.

### 3.2 Outcomes

Over a median follow‐up of 8.0 months, a total of 39 VTE were observed. The cumulative incidence of VTE at 1, 2 and 3 years was 5.0% (95% CI: 3.4–6.9), 9.0% (95% CI: 6.4–12.1) and 12.9% (95% CI: 7.7–19.5), respectively. During a median follow‐up for survival of 21.6 months, 258 deaths occurred. The median OS was 23.7 months (95% CI: 19.2–37.3), and the median PFS was 5.7 months (95% CI: 4.6–7.2). The overall response rate (ORR) was 31.3%, comprising 119 patients (21.8%) with partial response and 52 patients (9.5%) with complete remission. Stable disease was observed in 98 patients (17.9%), while 278 patients (50.8%) experienced primary disease progression.

### 3.3 Association Between Baseline Inflammatory Indices and Risk of VTE

In univariable analysis, the SHR for VTE per doubling of baseline levels was 1.29 (95% CI: 0.77–2.16) for NLR, 0.51 (95% CI: 0.24–1.11) for LMR, 1.24 (95% CI: 0.81–1.91) for PLR, 1.19 (95% CI: 0.87–1.64) for SII, and 1.15 (95% CI: 0.71–1.87) for CAR. In multivariable models adjusting for age, sex, tumour type, and stage, effect estimates remained similar, with SHRs of 1.42 (95% CI: 0.77–2.60) for NLR, 0.46 (95% CI: 0.19–1.211) for LMR, 1.40 (95% CI: 0.85–2.33) for PLR, 1.27 (95% CI: 0.88–1.83) for SII, and 1.13 (95% CI: 0.67–1.91) for CAR. Overall, baseline indices showed no strong associations with VTE risk, though higher LMR displayed a trend toward reduced risk.

### 3.4 Longitudinal Changes in Inflammatory Indices and Risk of VTE

We next examined dynamic changes by comparing patients with doubling of levels within the first 3 months of ICI therapy to those without. In univariable models, SHRs were 1.20 (95% CI: 0.42–3.40) for NLR, 2.53 (95% CI: 0.91–7.02) for PLR, 1.48 (95% CI: 0.55–4.01) for LMR, 1.32 (95% CI: 0.54–3.22) for SII, and 1.99 (95% CI: 0.88–4.46) for CAR. After multivariable adjustment, effect estimates remained similar: 1.15 (95% CI: 0.39–3.38) for NLR, 3.03 (95% CI: 1.05–8.80) for PLR, 1.59 (95% CI: 0.55–4.54) for LMR, 1.31 (95% CI: 0.52–3.29) for SII, and 2.02 (95% CI: 0.89–4.60) for CAR. In Table 2, the associations between inflammatory indices and VTE risk is summarized.

**TABLE 2:** Association of inflammatory indices with risk of VTE.

Inflammatory index	Baseline levels, continuous^a^—SHR (95% CI)		Doubling of levels within 3 months—SHR (95% CI)	
	Univariable	Adjusted	Univariable	Adjusted
NLR	1.29 (0.77–2.16), *p* = 0.341	1.42 (0.77–2.60), *p* = 0.258	1.20 (0.42–3.40), *p* = 0.731	1.15 (0.39–3.38), *p* = 0.794
PLR	1.24 (0.81–1.91), *p* = 0.324	1.40 (0.85–2.33), *p* = 0.189	2.53 (0.91–7.02), *p* = 0.074	3.03 (1.05–8.80), *p* = 0.041
LMR	0.51 (0.24–1.11), *p* = 0.088	0.46 (0.19–1.11), *p* = 0.083	1.48 (0.55–4.01), *p* = 0.435	1.59 (0.55–4.54), *p* = 0.386
SII	1.19 (0.87–1.64), *p* = 0.278	1.27 (0.88–1.83), *p* = 0.207	1.32 (0.54–3.22), *p* = 0.540	1.31 (0.52–3.29), *p* = 0.561
CAR	1.15 (0.71–1.87), *p* = 0.568	1.13 (0.67–1.91), *p* = 0.639	1.99 (0.88–4.46), *p* = 0.096	2.02 (0.89–4.60), *p* = 0.092

Abbreviations: CAR, C‐reactive protein‐to‐albumin ratio; CI, confidence interval; LMR, lymphocyte‐to‐monocyte ratio; NLR, neutrophil‐to‐lymphocyte ratio; PLR, plate‐let‐to‐lymphocyte ratio; SHR, sub‐distribution hazard ratio; SII, systemic immune‐inflammation index; VTE, venous thromboembolism.

^a^ Baseline levels analysed on log2‐scale (SHR per double increase in baseline levels). Multivariable adjustment for age, sex, cancer type, cancer stage, BMI, prior VTE, and baseline anticoagulation.

**FIGURE 1:**
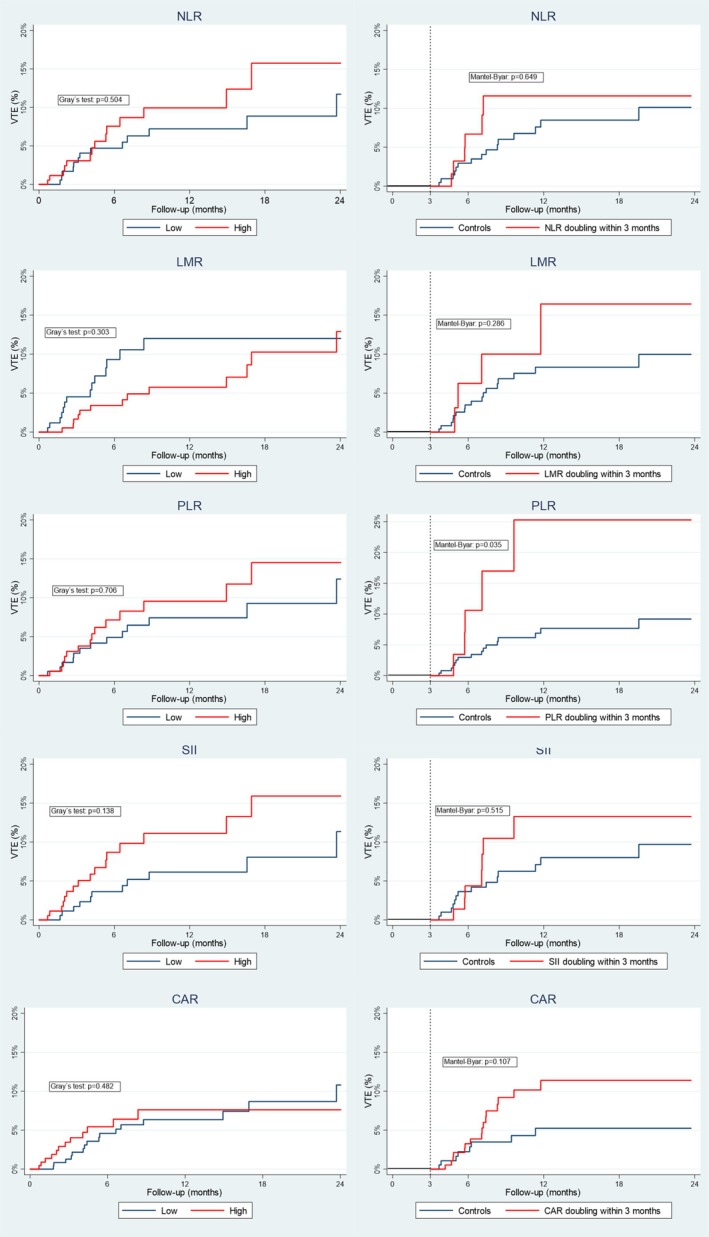
Cumulative incidence of VTE according to baseline and longitudinal values of inflammatory indices.

The 24‐month cumulative incidence of VTE in those with LMR doubling within 3 months of treatment start was 16.4% (95% CI: 6.1–40.1) compared to 9.5% (95% CI: 5.9–16.4) in those without (Mantel‐Byar: *p* = 0.286), 25.3% (95% CI: 10.5–53.6) with PLR doubling compared to 9.2% (95% CI: 5.4–15.4) without (Mantel‐Byar: *p* = 0.035), and 11.4% (95% CI: 7.0–18.3) with CAR‐doubling compared to 5.2% (95% CI: 2.6–10.4) in those without (Mantel‐Byar: *p* = 0.107). In Figure 1, cumulative VTE incidence estimates according to inflammatory indices at baseline and longitudinally within 3 months are displayed.

### 3.5 Association Between Baseline Inflammatory Indices and Treatment Response and Survival

In univariable analyses, higher baseline NLR was associated with shorter OS (HR per doubling 1.70, 95% CI: 1.40–2.07) and PFS (HR 1.29, 95% CI: 1.10–1.52). These associations remained significant after multivariable adjustment for age, sex, cancer type, stage, and treatment line (OS HR 1.65, 95% CI: 1.28–2.12; PFS HR 1.29, 95% CI: 1.05–1.57). PLR showed similar trends, with univariable associations for OS (HR 1.30, 95% CI: 1.09–1.56) and PFS (HR 1.22, 95% CI: 1.07–1.40), which were attenuated in multivariable models (OS HR 1.16, 95% CI: 0.92–1.45; PFS HR 1.18, 95% CI: 1.00–1.39). Higher levels of LMR corresponded to a reduced risk of death (OS HR 0.36, 95% CI: 0.26–0.50) and disease progression (PFS HR 0.55, 95% CI: 0.43–0.70), which was confirmed upon multi‐variable adjustment (OS HR 0.44, 95% CI: 0.30–0.65; PFS HR 0.57, 95% CI: 0.43–0.77). SII was modestly associated with worse OS (HR 1.25, 95% CI: 1.10–1.43) and PFS (HR 1.13, 95% CI: 1.02–1.26) in univariable models, with borderline significant associations after multivariable adjustment (OS HR 1.18, 95% CI: 1.00–1.39; PFS HR 1.12, 95% CI: 0.99–1.27). Higher CAR levels were associated with shorter OS, both in univariable analysis (HR 1.93, 95% CI: 1.67–2.24) and multivariable analysis (HR 1.99, 95% CI: 1.67–2.38), and with shorter PFS in univariable (HR 1.44, 95% CI: 1.28–1.62) and multi‐variable models (HR 1.42, 95% CI: 1.23–1.64).

**FIGURE 2:**
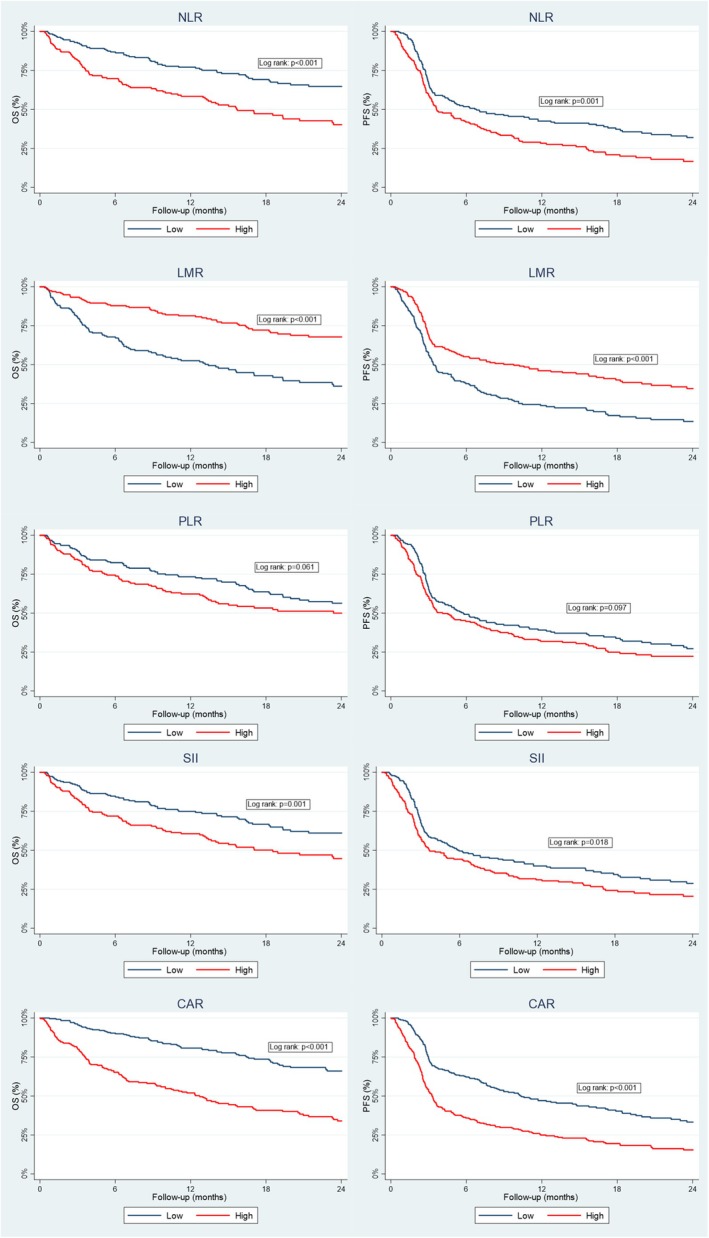
OS and PFS according to baseline inflammatory indices.

For DCR, lower baseline LMR was associated with greater odds of achieving disease control in both univariable (OR 1.85, 95% CI: 1.21–2.82) and multivariable analyses (OR 1.97, 95% CI: 1.21–3.20). Similarly, higher CAR was associated with lower odds for disease control in univariable analysis (OR: 0.53, 95% CI: 0.41–0.79) and multivariable analysis (OR: 0.51, 95% CI: 0.37–0.69). In contrast, higher baseline NLR (OR 0.79, 95% CI: 0.57–1.09), PLR (OR 0.84, 95% CI: 0.63–1.12), and SII (OR 0.88, 95% CI: 0.72–1.08) were not significantly associated with DCR after multivariable adjustment. In Table 3, associations between inflammatory indices and OS, PFS and DCR are summarized, and in Figure 2, OS and PFS estimates for dichotomized biomarkers levels at baseline are displayed.

**TABLE 3:** Association of inflammatory indices with OS and PFS.

Inflammatory index	OS (HR, 95% CI)		PFS (HR, 95% CI)		ORR (OR, 95% CI)	
	Univariable	Adjusted	Univariable	Adjusted	Univariable	Adjusted
NLR	1.70 (1.40–2.07), *p* < 0.001	1.65 (1.28–2.12), *p* < 0.001	1.29 (1.10–1.52), *p* = 0.002	1.29 (1.05–1.57), *p* = 0.013	0.79 (0.59–1.05), *p* = 0.101	0.79 (0.57–1.09), *p* = 0.146
PLR	1.30 (1.09–1.56), *p* = 0.004	1.16 (0.92–1.45), *p* = 0.202	1.22 (1.07–1.40), *p* = 0.004	1.18 (1.00–1.39), *p* = 0.048	0.82 (0.64–1.05), *p* = 0.122	0.84 (0.63–1.12), *p* = 0.237
LMR	0.36 (0.26–0.50), *p* < 0.001	0.44 (0.30–0.65), *p* < 0.001	0.55 (0.43–0.70), *p* < 0.001	0.57 (0.43–0.77), *p* < 0.001	1.85 (1.21–2.82), *p* = 0.004	1.97 (1.21–3.20), *p* = 0.222
SII	1.25 (1.10–1.43), *p* = 0.001	1.18 (1.00–1.39), *p* = 0.049	1.13 (1.02–1.26), *p* = 0.016	1.12 (0.99–1.27), *p* = 0.073	0.86 (0.72–1.03), *p* = 0.108	0.88 (0.72–1.08), *p* = 0.007
CAR	1.93 (1.67–2.24), *p* < 0.001	1.99 (1.67–2.38), *p* < 0.001	1.44 (1.28–1.62), *p* < 0.001	1.42 (1.23–1.64), *p* < 0.001	0.53 (0.41–0.70), *p* < 0.001	0.51 (0.37–0.69), *p* < 0.001

*Note:* All analyses conducted for baseline levels analysed on log2‐scale (SHR per double increase in baseline levels). Multivariable adjustment for age, sex, cancer type, stage, and line of systemic anticancer therapy.

Abbreviations: CAR, C‐reactive protein‐to‐albumin ratio; CI, confidence interval; HR, hazard ratio; LMR, lymphocyte‐to‐monocyte ratio; NLR, neutrophil‐to‐lymphocyte ratio; OR, odds ratio; ORR, overall response rate; OS, overall survival; PFS, progression‐free survival; PLR, platelet‐to‐lymphocyte ratio; SII, systemic immune‐inflammation index.

### 3.6 Longitudinal Changes in Inflammatory Indices and Progression‐ and Overall Survival

Patients with doubling of inflammatory indices during the first 3 months had shorter OS and PFS for certain markers. In univariable models, HRs for OS were 2.17 (95% CI: 1.50–3.14) for NLR, 1.87 (95% CI: 1.11–3.16) for PLR, 0.82 (95% CI: 0.49–1.37) for LMR, 1.82 (95% CI: 1.24–2.68) for SII, and 1.26 (95% CI: 0.95–1.67) for CAR. After multivariable adjustment, HRs were 2.34 (95% CI: 1.60–3.44) for NLR, 2.04 (95% CI: 1.20–3.46) for PLR, 0.86 (95% CI: 0.49–1.51) for LMR, 1.96 (95% CI: 1.32–2.89) for SII, and 1.29 (95% CI: 0.97–1.72) for CAR.

For PFS, in univariable analyses, the HR for disease progression in patients with increased levels was 1.62 (95% CI: 1.23–2.14) for NLR, 0.91 (95% CI: 0.61–1.34) for LMR, 1.42 (95% CI: 0.89–2.24) for PLR, 1.49 (95% CI: 1.12–1.99) for SII, and 1.36 (95% CI: 1.07–1.73) for CAR. After multivariable adjustment, effect estimates remained consistent, with HRs of 1.65 (95% CI: 1.26–2.16) for NLR, 0.93 (95% CI: 0.62–1.41) for LMR, 1.50 (95% CI: 0.94–2.39) for PLR, 1.52 (95% CI: 1.14–2.03) for SII, and 1.38 (95% CI: 1.09–1.74) for CAR, suggesting that early increases in NLR, PLR, SII, and CAR were associated with higher risk of disease progression and death (Table S2).

## 4 Discussion

In this single‐center cohort of patients with mostly advanced solid cancers treated with ICIs, we evaluated the associations of systemic inflammatory indices with VTE, treatment response, and survival. Baseline inflammatory markers showed no apparent associations with VTE risk, although higher LMR displayed a trend toward lower risk. In contrast, longitudinal analyses revealed that early increases in PLR and CAR within the first 3 months of treatment might be associated with a higher VTE risk. Regarding survival and treatment response, higher baseline levels of several inflammatory indices were associated with shorter PFS and OS, especially for NLR and CAR. Moreover, early rises in NLR, PLR, SII, and CAR during treatment were associated with increased risks of progression and death, indicating that on‐treatment changes in inflammatory indices may provide additional prognostic information beyond baseline values. Overall, these findings highlight the potential prognostic value of systemic inflammation in patients receiving ICIs, suggesting that baseline and longitudinal values might be associated with clinical outcomes.

Our findings confirm previous reports on the prognostic relevance of systemic inflammatory indices in patients with cancer. Several studies across cancer types have demonstrated robust associations between elevated baseline NLR, PLR, SII and CAR and lower levels of LMR with poor outcomes in patients receiving ICI [11, 15, 16, 17]. Consistent with these data, we observed that higher baseline NLR, PLR, SII, and particularly CAR were associated with shorter OS and PFS, while higher LMR was associated with improved outcomes. This observation aligns with the well‐established prognostic role of CRP‐ and albumin‐based scores such as the Glasgow Prognostic Score, as CAR is derived from the same biomarkers while reflecting systemic inflammation on a continuous scale [18]. We previously demonstrated that beyond baseline levels of inflammatory biomarkers, longitudinal changes in levels might increase their predictive utility. Specifically, longitudinal dynamics of CRP levels over the first 3 months of ICI therapy were identified as biomarker for shorter OS and PFS and a higher risk of VTE [19, 20]. Further, a prognostic role of longitudinal changes in inflammatory indices including NLR and PLR has been reported in ICI‐treated patients [21, 22, 23, 24]. Our study adds to the existing literature by showing that dynamic increases in inflammatory indices during ICI therapy were associated with inferior outcomes, suggesting that changes in inflammatory status during treatment may capture evolving immune or tumour dynamics that are associated with response to ICI therapy.

In contrast, data on inflammatory indices and VTE risk in ICI‐treated patients are limited, and currently no data is available evaluating longitudinal changes in inflammatory indices and risk of VTE. In the general cancer population, selected inflammatory indices including NLR and PLR have been linked to an increased VTE risk [25, 26]. While baseline indices were not associated with VTE in our cohort, early increases in PLR and CAR were associated with a trend toward higher VTE incidence. These findings support previous evidence that systemic inflammation and platelet activation play interrelated roles in cancer‐associated VTE, suggesting that inflammatory responses triggered or enhanced during ICI therapy may potentiate thrombo‐inflammatory mechanisms in patients [6, 8, 27].

The clinical relevance of our findings lies primarily in their potential utility for risk stratification. Inflammatory indices such as NLR, PLR, LMR, SII, and CAR are inexpensive, routinely available laboratory parameters that can be readily assessed in clinical practice. While these biomarkers are not currently used to guide treatment decisions, their association with clinical outcomes suggests that they may help identify patients at increased risk of poor outcomes who could benefit from closer clinical monitoring, earlier reassessment of treatment response, or inclusion in risk‐adapted clinical strategies. Furthermore, given that some inflammatory markers, particularly NLR, have demonstrated prognostic and predictive value in other cancer settings and have been incorporated as stratification factors in clinical trials [28], our findings support further investigation of these indices in patients treated with ICI, yet should be considered hypothesis‐generating and warrant confirmation in independent, prospective, and ideally multi‐center studies before clinical implementation, specifically to define optimal timing, monitoring intervals, and potential thresholds for inflammatory indices.

The observed associations between inflammatory indices and clinical outcomes may be explained by the role of systemic inflammation in modulating tumour immunity and haemostatic pathways alike. Elevated neutrophil‐ and platelet‐based markers reflect a tumour‐promoting inflammatory environment characterized by immunosuppressive myeloid activation, cytokine release, and impaired cytotoxic lymphocyte function, which may attenuate ICI efficacy [29, 30, 31]. Conversely, higher LMR indicates an increased lymphocyte‐mediated immune response, which might indicate enhanced anti‐tumoral immunity [11]. Regarding VTE risk, systemic inflammation promotes endothelial activation, platelet aggregation, and tissue factor expression, jointly contributing to a prothrombotic state [27, 32, 33]. Experimental data have indicated that ICI might promote a prothrombotic state via increased tissue factor expression on tumour cells and inflammatory cells of the tumour‐microenvironment, higher levels of pro‐thrombotic neutrophil‐extracellular traps and circulating neutrophil–platelet‐aggregates [34, 35]. Therefore, inflammatory indices may capture both tumour‐related immune dysregulation associated with cancer progression and systemic inflammation that reflects ICI‐related prothrombotic risk. Emerging clinical evidence suggests that dynamic changes in systemic inflammatory indices during ICI therapy may aid in differentiating true progression from pseudo‐progression, under‐scoring their potential as dynamic biomarkers of treatment response [36]. Collectively, these observations support a link between longitudinal inflammatory dynamics, immune modulation, and thrombo‐inflammatory processes during immunotherapy.

Several limitations of our study should be acknowledged. First, its retrospective, single‐center design may have introduced selection bias and limits the generalizability of the findings. Further, due to the retrospective design, additional biomarkers including D‐dimer and PD‐L1 expression were not routinely available and could therefore not be included in the multivariable analyses. Second, although the overall cohort was relatively large, the number of outcome events in the VTE analyses was limited, potentially reducing statistical power. Therefore, analyses regarding VTE risk have to be interpreted with caution, considering the potential for false positive or false negative findings. Third, inflammatory markers were assessed at routine clinical time points rather than standardized intervals, which may have introduced variability in the measurements. Fourth, based on the observational nature of this study, residual confounding cannot be excluded. Although analyses were adjusted for established prognostic factors and outcome‐specific confounders, unmeasured or incompletely measured variables, including specific prior anticancer treatments, comorbidities, and other patient‐ or disease‐related characteristics, may have contributed to the observed associations. Importantly, detailed information on active infections or chronic inflammatory conditions at treatment initiation and longitudinally was not systematically available and could therefore not be accounted for, also introducing the potential for residual confounding. Fifth, heterogeneity in tumour types, prior therapies, and ICI regimens may have influenced both inflammatory responses and clinical outcomes. Sixth, the study population may overrepresent patients with better performance status, as patients treated at tertiary cancer centers and selected for ICI therapy are generally required to be fit for treatment. However, indicators such as age distribution, comorbidity burden, and ECOG performance status suggest that the cohort is broadly representative of consecutively treated patients in routine clinical practice. Further, based on the observational nature of this study, informative censoring cannot be excluded. Given the substantial variability in treatment duration and follow‐up, treatment discontinuation or censoring may have been related to disease status, treatment response, or other clinical factors, which could have influenced the observed associations. Finally, no formal adjustment for multiple testing was performed. Given the exploratory nature of the analyses and the evaluation of multiple inflammatory biomarkers across several outcomes, the observed associations should be interpreted with caution, as some findings may represent chance observations. Consequently, our results should be considered hypothesis‐generating and warrant validation in independent cohorts.

In conclusion, selected baseline inflammatory indices might be associated with survival and treatment response in patients treated with ICIs, while early longitudinal changes in PLR and CAR may be indicative of an increased risk of VTE. Markers such as NLR, PLR, LMR, SII, and CAR may serve as easily accessible, cost‐effective biomarkers, helping to identify patients at higher risk of disease progression, death, or VTE. These findings support further prospective validation and suggest that integrating inflammatory indices into clinical assessment could improve individualized management of patients receiving ICI.

## Author Contributions

Conceptualization, project administration, supervision, writing – original draft, L.A.; Project administration, writing – original draft, I.P.; Writing – review and editing, M.P.; Writing – review and editing, A.S.B.; Writing – review and editing, C.H.; Conceptualization, project administration, supervision, writing – review and editing, C.A. Conceptualization, data curation, formal analysis, methodology, project administration, supervision, writing – original draft, F.M.; All authors have read and approved the submission of the manuscript.

## Funding

This work was supported by funding obtained by F.M. with the Early Career Research Grant of the “Gesellschaft für Thrombose‐ und Hämostaseforschung” (GTH) 2021 (Society for Thrombosis and Haemostasis Research) and the Start Funding Program 2024 (Medical University of Graz). Furthermore, the financial support by the Christian Doppler Research Association is gratefully acknowledged.

## Conflicts of Interest

L.A. has no conflicts of interest to declare. I.P. has no conflicts of interest to declare. M.P. has received honoraria for lectures, consultation, or advisory board participation from the following for‐profit companies: Bayer, Bristol Myers Squibb, Novartis, Gerson Lehrman Group (GLG), CMC Contrast, GlaxoSmithKline, Mundipharma, Roche, BMJ Journals, MedMedia, Astra‐Zeneca, AbbVie, Lilly, Medahead, Daiichi Sankyo, Sanofi, Merck Sharp & Dohme, Tocagen, Adastra, Gan and Lee Pharmaceuticals, Janssen, Servier, Miltenyi, Böhringer‐Ingelheim, Telix, Medscape, OncLive, Medac, Nerviano Medical Sciences, and ITM Oncologics GmbH. A.S.B. has received research support from Daiichi Sankyo and Roche, honoraria for lectures, consultation, or advisory board participation from Roche, Bristol Myers Squibb, Merck, Daiichi Sankyo, Astra‐Zeneca, CeCaVa, and Seagen, and travel support from Roche, Amgen. C.H. has received speaker honoraria from Amgen, BMS, MSD, Novartis, and Roche; and participated on advisory boards for Amgen, Astra Zeneca, BMS, Inzyte, MSD, Novartis, Pierre Fabre, and Roche. C.A. has received personal fees for lectures and/or participation in advisory boards from Bayer, Daiichi Sankyo, BMS/Pfizer alliance, and Sanofi. F.M. has received travel/congress support from Novartis and Bayer, honoraria for lectures from Servier and Bristol Myers Squibb, honoraria for consulting from Johnson and Johnson, and participated in advisory boards for MSD, Merck, and Servier.

## Supporting information


**Table S1:** Levels of inflammatory indices.
**Table S2:** Association between longitudinal changes of inflammatory indices and OS/PFS.

File S1

## Data Availability

The datasets generated during and/or analysed during the current study are available from the corresponding author on reasonable request.
