# Supplementary material for: Systemic Inflammation and Risk of Thromboembolism, Mortality, and Treatment Response in Patients With Cancer Receiving Immune Checkpoint Inhibitors
Source: Eur J Clin Invest. 2026 Jul 24;56(8):e70243. doi: 10.1111/eci.70243 (PMC13397391; doi:10.1111/eci.70243)
Supplement: Supplementary file 1 — Table S1: Levels of inflammatory indices. Table S2: Association between longitudinal changes of inflammatory indices and OS/PFS. [file ECI-56-e70243-s001.docx]

## Supplemental Table 1: Levels of inflammatory indices.

| **Inflammatory index** | **Median (IQR) - Baseline** | **Median (IQR) – within 3 months** | **Patients with doubling within 3 months (n, %)** |
| --- | --- | --- | --- |
| NLR | 4.0 (2.6-6.0) | 3.8 (2.4 – 6.3) | 146 (25.2%) |
| PLR | 220.5 (154.8-319.2) | 202.6 (139.7 – 302.7) | 64 (11.0%) |
| LMR | 2.0 (1.3-2.8) | 1.9 (1.3 – 2.9) | 70 (12.1%) |
| SII | 1,010.8 (611.0-1,779.4) | 941.5 (564.3 – 1723.1) | 165 (28.4%) |
| CAR | 0.33 (0.09-1.19) | 0.36 (0.09 – 1.53) | 297 (51.2%) |

**Abbreviations**: CAR, C-reactive protein-to-albumin ratio; IQR, interquartile range; LMR, lymphocyte-to-monocyte ratio; NLR, neutrophil-to-lymphocyte ratio; PLR, platelet-to-lymphocyte ratio; SII, systemic immune-inflammation index.

## Supplemental Table 2: Association between longitudinal changes of inflammatory indices and OS/PFS.

| **Inflammatory index** | **OS - SHR for death for doubling of levels within 3 months (95%CI)** | | **PFS - SHR for death for doubling of levels within 3 months (95%CI)** | |
| --- | --- | --- | --- | --- |
|  | **Univariable** | **Adjusted** | **Univariable** | **Adjusted** |
| NLR | 2.17 (1.50–3.14), p<0.001 | 2.34 (1.60–3.44), p<0.001 | 1.62 (1.23–2.14), p=0.001 | 1.65 (1.26–2.16), p<0.001 |
| PLR | 1.87 (1.11–3.16), p=0.020 | 2.04 (1.20–3.46), p=0.009 | 1.42 (0.89–2.24), p=0.137 | 1.50 (0.94–2.39), p=0.088 |
| LMR | 0.82 (0.49–1.37), p=0.448 | 0.86 (0.49–1.51), p=0.604 | 0.91 (0.61–1.34), p=0.615 | 0.93 (0.62–1.41), p=0.734 |
| SII | 1.82 (1.24–2.68), p=0.003 | 1.96 (1.32–2.89), p=0.001 | 1.49 (1.12–1.99), p=0.007 | 1.52 (1.14–2.03), p=0.005 |
| CAR | 1.26 (0.95–1.67), p=0.113 | 1.29 (0.97–1.72), p=0.077 | 1.36 (1.07–1.73), p=0.013 | 1.38 (1.09–1.74), p=0.008 |

**Table footnote**: Multivariable adjustment for age, sex, cancer type, stage, and line of systemic anticancer therapy. Abbreviations: CAR, C-reactive protein-to-albumin ratio; CI, confidence interval; HR, hazard ratio; LMR, lymphocyte-to-monocyte ratio; NLR, neutrophil-to-lymphocyte ratio; OS, overall survival; PFS, progression-free survival; PLR, platelet-to-lymphocyte ratio; SII, systemic immune-inflammation index.
